# Design and Fabrication of a High-Temperature SOI Pressure Sensor with Optimized Crossbeam Membrane

**DOI:** 10.3390/mi14051045

**Published:** 2023-05-12

**Authors:** Le Hao, Cun Li, Lukang Wang, Bing Bai, Yulong Zhao, Chao Luo

**Affiliations:** 1 State Key Laboratory for Manufacturing Systems Engineering, Xi’an Jiaotong University, Xi’an 710049, China; hlxidian@163.com (L.H.); wanglukang@stu.xjtu.edu.cn (L.W.); bingbai@xjtu.edu.cn (B.B.); zhaoyulong@xjtu.edu.cn (Y.Z.); 2 Xi’an Chinastar M&C Ltd., Xi’an 710077, China; lc@websensor.com

**Keywords:** SOI pressure sensor, high accuracy, high temperature lead

## Abstract

This paper presents a SOI piezoresistive pressure sensor with the crossbeam membrane. The roots of the crossbeam were widened, which solved the problem of the poor dynamic performance of small-range pressure sensors working at a high temperature of 200 °C. A theoretical model was established to optimize the proposed structure, which combined the finite element and the curve fitting. Using the theoretical model, the structural dimensions were optimized to obtain the optimal sensitivity. During optimization, the sensor nonlinearity was also taken into consideration. The sensor chip was fabricated by MEMS bulk-micromachining technology, and Ti/Pt/Au metal leads were prepared to improve the sensor ability of high-temperature resistance over a long time. The sensor chip was packaged and tested, and the experimental results show the sensor achieved an accuracy of 0.241% FS, nonlinearity of 0.180% FS, hysteresis of 0.086% FS and repeatability of 0.137% FS at the high temperature. Given the good reliability and performance at the high temperature, the proposed sensor provides a suitable alternative for the measurement of pressure at high temperatures.

## 1. Introduction

As pressure measurement in high-temperature environments becomes more common, high-temperature pressure sensors have received increasing attention from researchers. The sensing mechanisms of pressure sensors primarily include the piezoresistive effect, piezoelectric effect, resonance sensing and capacitive sensing, while pressure sensors based on piezoresistive effect have the advantages of good performance and the direct signal transduction mechanism [1,2]. To solve the problem of conventional silicon piezoresistive pressure sensors generating leakage currents at high temperatures [3,4], new materials such as SiC, diamond and SOI have been extensively investigated [2,5,6]. Notably, SOI has the advantages of easy preparation and low cost [7,8]. Therefore, SOI high-temperature piezoresistive pressure sensors are in increasing demand for applications in automotives [9], aerospace [10], petroleum [11], etc.

Low sensitivity and poor output stability are two important problems with current SOI high-temperature piezoresistive pressure sensors. The reason for the low sensitivity is that the piezoresistor is heavily doped to reduce the drift caused by high temperatures, thus making the piezoresistive coefficient of the sensor very low. The sensitivity depends mainly on the piezoresistive coefficient and the stress at the piezoresistor’s placement. Therefore, the only way to increase the sensitivity while keeping the temperature drift small is to increase the stress on the sensitive membrane of the pressure sensor as much as possible. For the flat membrane, the stress is increased mainly by adjusting the size of the membrane. Xu et al. designed a pressure sensor with a pressure range of 30 kPa and a square membrane of 700 µm × 700 µm × 2.1 µm, but with a large repeatability error [12]. Song et al. developed a pressure sensor with a pressure range of 180 kPa and a square membrane of 100 µm × 100 µm × 2 µm, but with a large hysteresis error [13]. Zhao et al. proposed a pressure sensor with a pressure range of 100 kPa and a square membrane of 1000 µm × 1000 µm [14]. Hase et al. proposed a pressure sensor with a pressure range of 100 kPa and a circular membrane with a diameter of 1.5 mm, but with a large nonlinearity error [15]. Chung et al. developed a pressure sensor with a pressure range of 700 mmHg featuring a rectangular membrane of 360 μm × 1140 μm × 5 μm [16]. It can be seen that for small-range pressure sensors, the thickness of the flat membrane is small, which leads to a reduction in the stiffness of the sensitive membrane, reduces the response frequency and makes processing very difficult. For the non-flat membrane, the stress concentration region is created by designing special structures to increase the stress on the membrane. Li et al. designed a novel piezoresistive pressure sensor with a four-grooved membrane combined with a rood beam for measuring pressures of less than 1 psi [17]. Xu et al. developed a novel bossed diaphragm combined with a peninsula-island structure with a pressure range of 500 Pa, but with a large nonlinearity error [18]. Meng et al. developed a piezoresistive pressure sensor with a beam-membrane-dual-island structure for micro-pressure monitoring [19]. By designing several structures on the pressure sensitive membrane, the stress concentration can be effectively generated, but it is difficult to fabricate.

An important reason for poor output stability is that it is difficult to fabricate metal leads that work reliably at high temperatures. Xu et al. prepared an AlCu electrode that was capable of operating at 160 °C [12]. Tang et al. prepared a Ti/TiN/Pt/Au multilayer lead, but the accuracy of the sensor was poor [20]. Li et al. developed a Cr/Au multilayer lead, which was suitable for 150 °C [17]. Wang et al. deposited a Pt5Si2/Ti/Pt/Au multilayer lead, which was able to operate at 220 °C, but the sensitivity was low [21]. Although extensive research has been performed on metal leads, few researchers have verified the reliability of the leads at high temperatures over a long time.

This paper introduces a SOI piezoresistive pressure sensor with optimized crossbeam membrane. The characteristic frequency of the sensor chip was analyzed by the COMSOL Multiphysics software. By combining the finite element and curve fitting, the theoretical equations for the maximum transverse and longitudinal stress difference (MTALSD) and the maximum displacement (MD) with respect to the structural dimension were determined. Finally, the optimum value of the structural dimension was obtained by an optimized solution in MATLAB. The sensor chip was fabricated, packaged and tested, and then long-term reliability testing of the leads at high temperatures, as well as the static performance testing of the proposed sensor, were completed.

## 2. Structure Design and Optimization

### 2.1. Structure Design

**Figure 1:**
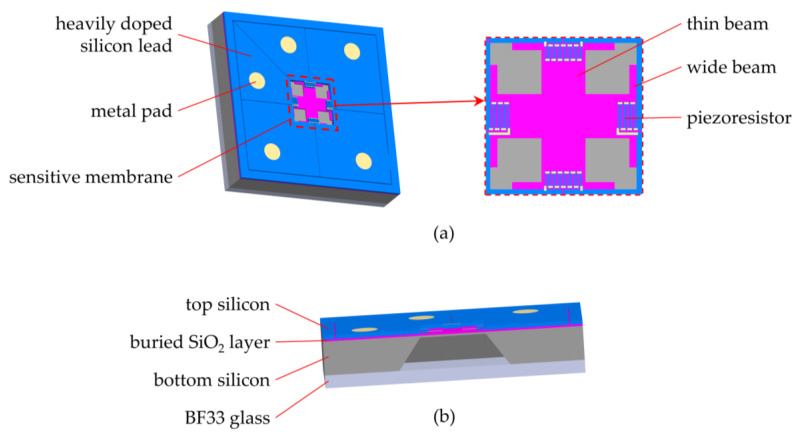
(**a**) The front side of the proposed sensor structure. (**b**) The section view of the proposed sensor structure.

In order to achieve pressure measurement at high temperatures, SOI was chosen as the substrate material. The pressure sensor chip consists of the top silicon, the buried SiO_2_ layer, the bottom silicon and the BF33 glass with a thermal expansion coefficient similar to silicon, as shown in Figure 1b. The top silicon is mainly used to fabricate piezoresistors, the buried SiO_2_ layer is used to achieve electrical insulation at high temperatures, the bottom silicon is mainly used to fabricate the cavity and the BF33 glass is used to form a sealed vacuum cavity with the SOI wafer. The metal pads, heavily doped silicon leads and the pressure sensitive membrane are shown in Figure 1a. The sensitive membrane is composed of the buried oxygen layer, and the structure in the bottom silicon. For ease of processing, heavily doped silicon leads were chosen for the connection of the piezoresistors to build the Wheatstone bridge [22].

**Figure 2:**
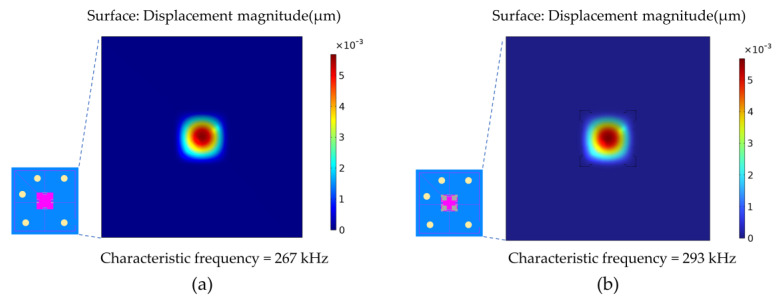
(**a**) The vibration mode of the square membrane. (**b**) The vibration mode of the root-widened crossbeam membrane.

On the whole, compared with flat and composite structural membranes, the beam membrane is easier to fabricate, has a higher characteristic frequency and has a better stress concentration effect. Therefore, the crossbeam structure with widened roots was designed by optimizing on the basis of previous studies [23,24]. The crossbeam is located on the front side of the pressure sensitive membrane, and the symmetry axis of the crossbeam is consistent with that of the sensor chip. When subjected to a forward pressure load, the stress concentration occurs in the root of the crossbeam, which can improve the sensitivity of the sensor. The crossbeam also strengthens the pressure sensitive membrane, increasing its stiffness and characteristic frequency. The characteristic frequency of the square membrane and the root-widened crossbeam membrane were analyzed by the COMSOL Multiphysics software, as shown in Figure 2. The main conditions of the simulation included: (1) The membrane thickness and the membrane length of the two kinds of membranes were the same. (2) The fixed constraint was applied on the back of the sensor chip. The main assumptions of the simulation included: (1) Compared to the sensitive membrane, structures such as piezoresistors and leads have little effect on the characteristic frequency and were therefore not considered. (2) The thickness of the buried oxygen layer is negligible compared to the thickness of the sensitive membrane. It can be seen from Figure 2 that the characteristic frequency of the root-widened crossbeam membrane is 26 kHz higher than that of the square membrane, which enables the proposed sensor to measure rapidly changing signals.

The wide beam at the root of the crossbeam makes the stress change in the stress concentration region smoother, which improves the performance of the sensor and the overload capacity of the sensor. Figure 1a shows the serpentine-shaped configuration of the transverse and longitudinal piezoresistors, which increases the resistance value of the piezoresistors. The metal layer was deposited at the corners of serpentine-shaped piezoresistors to reduce the effect of the longitudinal piezoresistive coefficient. The piezoresistors were all located in the wide beam section at the root of the crossbeam in order to obtain maximum stress.

### 2.2. Geometry Optimization

**Figure 3:**
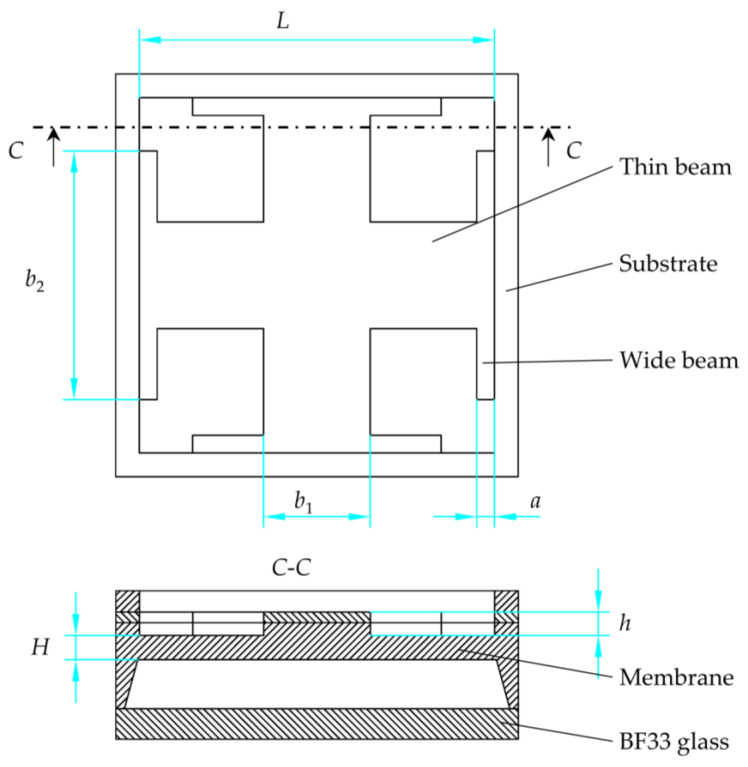
The definition of structural dimensions of the proposed chip.

In order to optimize the structural dimensions, the corresponding variables need to be defined for each structural dimension. *L* is the membrane length, *H* is the membrane thickness, *b*_1_ is the thin beam width, *b*_2_ is the wide beam length, *a* is the wide beam width and *h* is the beam thickness, as shown in Figure 3. The schematic of the sensor structure in Figure 3 looks different from the actual structure because drawing the schematic based on the actual dimensions results in the structure of the pressure sensitive membrane looking very small and thus not being able to mark the corresponding dimensions. The actual sensor structure was therefore simplified, and some dimensions were adjusted in order to clearly show the dimensions of the sensor structure.

The sensitivity and nonlinearity are two important indicators of pressure sensors, which are largely dependent on the structural dimension. The MTALSD on the membrane centerline is related to the sensitivity of the sensor, and the MD of the membrane center is related to the nonlinearity of the sensor [25]. Therefore, MTALSD and MD are important considerations when optimizing the structural dimension. In this paper, the optimum structural dimension was determined by solving the optimal model. In order to achieve the highest sensitivity, MTALSD was used as the objective function of the optimal model. Low nonlinearity is achieved when the MD is less than one-fifth of the membrane thickness [26]; hence, MD was used as the constraint function. The objective and constraint functions regarding the structural dimension then need to be determined separately by means of curve fitting. Before curve fitting, it is necessary to analyze whether each structural dimension variable has an effect on the MTALSD and MD. If a structural dimension has an effect on the MTALSD or MD, then it should only be considered when curve fitting for MTALSD or MD. Otherwise, the process of curve fitting cannot be completed, or the function determined by curve fitting is highly inaccurate.

**Figure 4:**
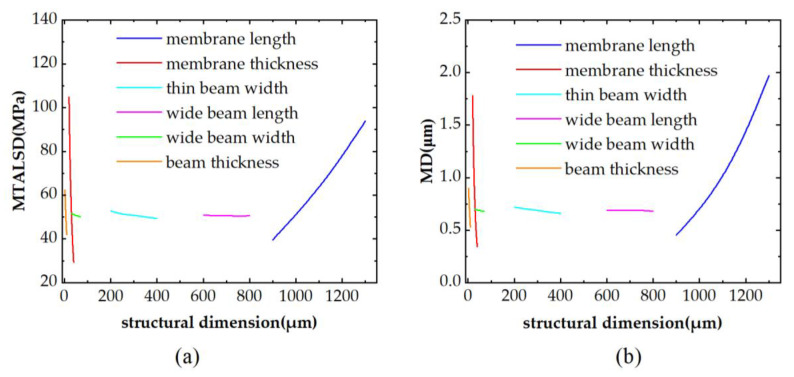
(**a**) The effects of structural dimensions on the MTALSD. (**b**) The effects of structural dimensions on the MD.

When analyzing the effect of a structural dimension on MTALSD and MD, this structural dimension takes a series of values within its scope, and the remaining structural dimensions take fixed values within their scopes. The MTALSD and MD regarding this structural dimension are then calculated by the COMSOL Multiphysics software. The effects of the membrane length *L*, the membrane thickness *H*, the thin beam width *b*_1_, the wide beam length *b*_2_, the wide beam width *a* and the beam thickness *h* on MTALSD on the centerline of the membrane and MD at the center of the membrane are shown in Figure 4. It can be seen that the membrane length *L*, the membrane thickness *H* and the beam thickness *h* have a large influence on the MTALSD. The thin beam width *b*_1_ and the wide beam width *a* have small effects on the MTALSD. The wide beam length *b*_2_ has almost no effect on the MTALSD. The membrane length *L*, the membrane thickness *H* and the beam thickness *h* have great effects on the MD. The thin beam width *b*_1_, the wide beam length *b*_2_ and the wide beam width *a* have almost no effect on the MD. As the wide beam length *b*_2_ has essentially no effect on both MTALSD and MD, the wide beam length *b*_2_ was fixed to 700 μm in this study.

The wide beam length *b*_2_ has essentially no effect on the MTALSD, and the thin beam width *b*_1_, wide beam length *b*_2_ and wide beam width *a* have essentially no effect on the MD. It is therefore not necessary to consider the wide beam length *b*_2_ when determining the function for MTALSD. It is not necessary to consider the thin beam width *b*_1_, the wide beam length *b*_2_ and the wide beam width *a* when determining the functions for MD. Therefore, based on the stress and deflection as functions of structural dimensions in a C-type membrane [17], the functional forms are followed by:

(1) $$\sigma =K_{1}\cdot L^{c1}\cdot H^{d1}\cdot b_{1}{}^{e1}\cdot a^{f1}\cdot h^{g1}$$

(2) $$\omega =K_{2}\cdot L^{c2}\cdot H^{d2}\cdot h^{g2}$$

where σ is MTALSD on the centerline of the membrane; ω is MD of the membrane center; *L*, *H*, *b*_1_, *a* and *h* are the structural dimensions as show in Figure 3; and *c*_1_, *d*_1_, *e*_1_, *f*_1_, *g*_1_, *c*_1_, *d*_2_, *g*_2_, *K*_1_ and *K*_2_ are the curve fitting coefficients.

In order to calculate the coefficients for all the structural dimensional variables in Equations (1) and (2), each dimensional variable needs to be discussed separately using the control variable method. When determining the coefficients corresponding to the membrane length *L*, Equations (1) and (2) can be written as:

(3) $$\sigma =K_{1L}\cdot L^{c1}$$

(4) $$\omega =K_{2L}\cdot L^{c2}$$

*K*_1*L*_ and *K*_2*L*_ are coefficients of the variable *L*. The other coefficients were mentioned before. The values of *σ* and *ω* with respect to a series of the variable *L* were then calculated by the COMSOL Multiphysics software, and then the power function was fitted by the curve-fitting toolbox in MATLAB. Then, Equations (3) and (4) are followed by:

(5) $$\sigma =4.642\times 10^{-6}L^{2.346}$$

(6) $$\omega =5.871\times 10^{-13}L^{4.022}$$

In the same way, MTALSD and MD can be obtained as functions of the membrane thickness *H* and the beam thickness *h*. They are derived as:

(7) $$\sigma =2.465\times 10^{4}H^{-1.821}$$

(8) $$\omega =2108H^{-2.362}$$

(9) $$\sigma =74.65h^{-0.2316}$$

(10) $$\omega =1.136h^{-0.3022}$$

Since the thin beam width *b*_1_ and the wide beam width *a* have essentially no effect on MD, the equations related to them are shown as:

(11) $$\sigma =86.06b_{1}{}^{-0.09269}$$

(12) $$\sigma =58.64a^{-0.03717}$$

The coefficients in Equations (3)–(12) were substituted into Equations (1) and (2). Equations (1) and (2) are listed as:

(13) $$\sigma =K_{1}\frac{L^{2.346}}{H^{1.821}a^{0.0371}b_{1}{}^{0.0926}h^{0.2316}}$$

(14) $$\omega =K_{2}\frac{L^{4.022}}{H^{2.362}h^{0.302}}$$

A set of dimensional variable values were taken for simulation to obtain the corresponding values of the MTALSD and MD. Then, substituting them into Equations (13) and (14), the values of *K*_1_ and *K*_2_ can be deduced to be 0.0068 and 3.1391 × 10^−9^, respectively. Thus, Equations (13) and (14) can be expressed as:

(15) $$\sigma =0.0068\frac{L^{2.346}}{H^{1.821}a^{0.0371}b_{1}{}^{0.0926}h^{0.2316}}$$

(16) $$\omega =3.1391\times 10^{-9}\frac{L^{4.022}}{H^{2.362}h^{0.302}}$$

From Equations (15) and (16), it can be found that the membrane length *L* has the greatest effect on *σ* and *ω*, followed by the membrane thickness *H*. The relevant dimensions of the membrane have greater effects on *σ* and *ω* than the relevant dimensions of the beam. Since the thin beam width *b*_1_ and the wide beam width *a* are not related in Equation (16), the value of *σ* can be increased by appropriately reducing their values, thus increasing the sensitivity.

MTALSD *σ* is taken as the objective function for optimization, and MD *ω* is taken as the constraint function, while other conditions are combined to establish the corresponding optimization model. MATLAB can then be used to find an optimal set of dimensional variable values, which can then be fine-adjusted to obtain the final dimensional variable values, as shown in Table 1. Substituting the final dimensional variable values into Equations (15) and (16), MTALSD is 98.52 MPa and MD is 1.76 μm. MD is less than one-fifth of the membrane thickness, satisfying the small deformation theory.

## 3. Fabrication and Package

Traditional aluminum leads are likely to fail at high temperatures. Therefore, a multilayer metal lead was developed in this paper, namely the contact silicon layer, the barrier diffusion layer and the gold wire bonding layer. The contact silicon layer was made of titanium, which, due to its high electrical conductivity and low contact resistance, does not degrade in performance at high temperatures or high current densities and is easy to deposit. The barrier diffusion layer was platinum, which is inactive and does not form undesired compounds with the metal it is in contact with. The gold wire bonding layer was made of gold due to its ease of soldering to the gold wire.

**Figure 5:**
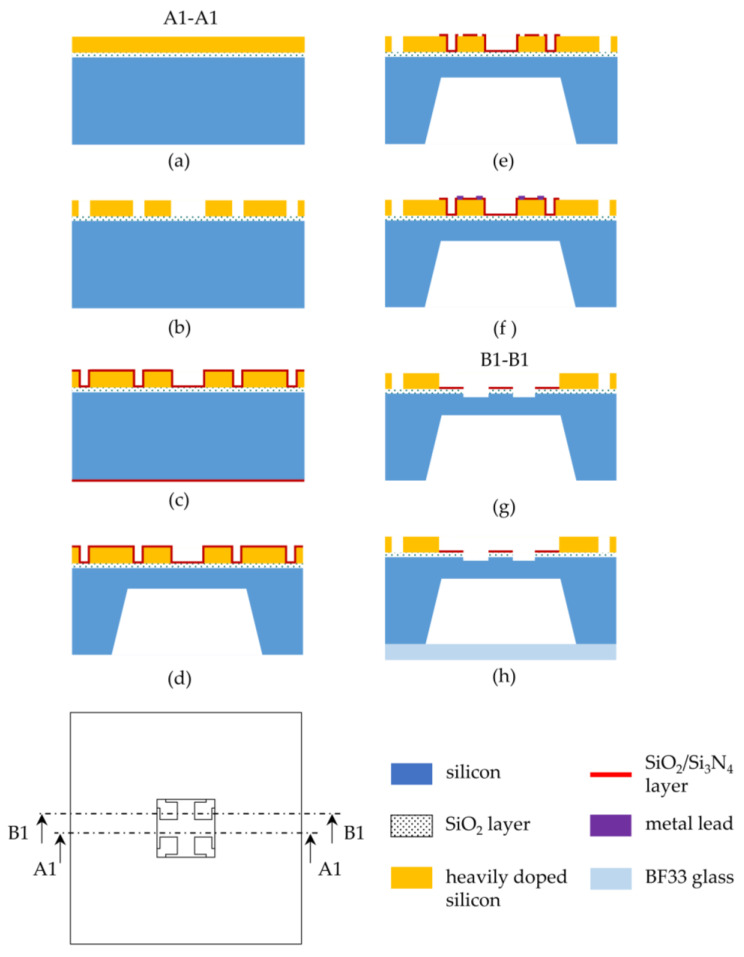
Sensor chip fabrication process: (**a**) implanting boron; (**b**) forming the piezoresistors and silicon leads; (**c**) depositing the SiO_2_/Si_3_N_4_ layers; (**d**) etching the cavity on the back side; (**e**) etching off the SiO_2_/Si_3_N_4_ layers; (**f**) depositing metal leads; (**g**) forming the crossbeam with widened roots; (**h**) anodic bonding.

A 4-inch P-type SOI wafer in the (100) direction was used and then fabricated by bulk-micromachining technology. An overview of the fabrication process of the sensor is shown in Figure 5.

(a) The SiO_2_ layers were grown on both sides of the wafer at 1100 °C in a high-temperature oxidation oven. Boron implantation was carried out on the front side with a dose of 1.25 × 10^16^ and an energy of 80 Kev, followed by holding at 1000 °C for 60 min for annealing.

(b) The front side was then etched using inductively coupled plasma (ICP) to form the piezoresistors and silicon leads. The top silicon of the sensitive membrane region was completely etched out.

(c) The SiO_2_/Si_3_N_4_ layers (SiO_2_ 150 nm, Si_3_N_4_ 100 nm) were deposited using low-pressure chemical vapor deposition (LPCVD) on the front and back sides of the wafer.

(d) Reactive ion etching (RIE) was used to etch the SiO_2_/Si_3_N_4_ layer in the central area of the back side of the wafer, and the remaining area of the SiO_2_/Si_3_N_4_ layer was used as a mask for etching the cavity. The cavity was then etched with KOH at a constant temperature. After the cavity was completed, the remaining SiO_2_/Si_3_N_4_ layer on the back side was removed.

(e) RIE was used to etch off the SiO_2_/Si_3_N_4_ layer at the corners of the piezoresistors and outside the pressure sensitive membrane.

(f) The Ti/Pt/Au films were deposited and patterned to form metal leads at the corners of the piezoresistors and metal pads. Ohmic contact was achieved by holding for 30 min at 550–700 °C in N_2_ ambient.

(g) The SiO_2_/Si_3_N_4_ layer, the partial regions of the buried oxygen layer and the bottom silicon on the front side were etched by RIE to form the crossbeam with widened roots.

(h) The anodic bonding of the SOI wafer to the glass was then completed under vacuum.

**Figure 6:**
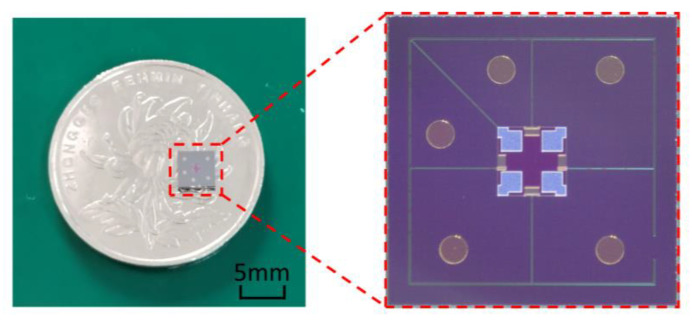
The fabricated pressure sensor chip.

The fabricated pressure sensor chip is shown in Figure 6, which indicates that the chip was relatively small. The crossbeam with widened roots was consistent with the design, and the metal pads were also intact. Thus, it can be demonstrated that the entire fabrication process of the chip is reasonable.

**Figure 7:**
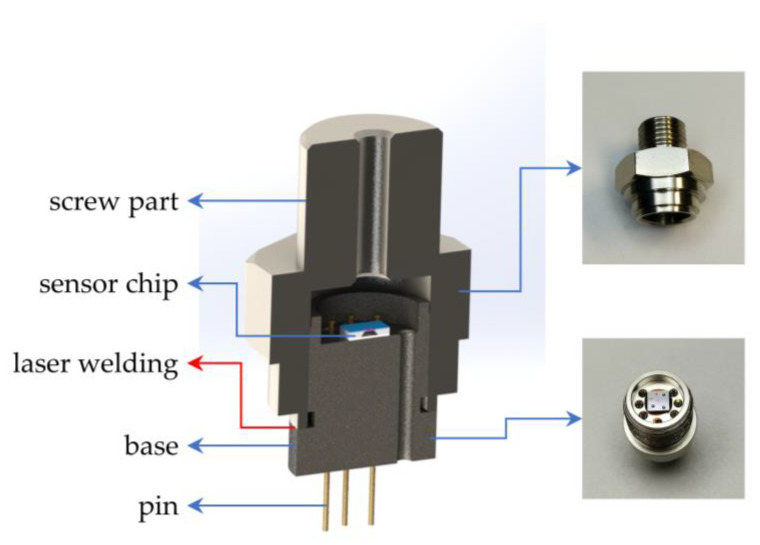
Package of the proposed sensor.

Once the pressure sensor chip is fabricated, it needs to be packaged before it can be tested and used subsequently. The package of the pressure sensor chip is shown in Figure 7. Both the base and the screw part were made of 316 L stainless steel, which improved the temperature resistance and corrosion resistance of the sensor. There were six pins on the base, five of which were used to lead out the electrical signals on the chip and one as a backup. The surface of these pins was coated with gold in order to facilitate the subsequent soldering of the gold wire. The screw part was machined with standard threads for mounting on the test equipment. The sensor chip was attached to the base with high-temperature resistant glue, and then the metal pads on the sensor chip were connected to the pins on the base with gold wires. The base with the sensor chip attached was then assembled to the screw part and sealed by laser welding.

## 4. Testing and Analyzing

**Figure 8:**
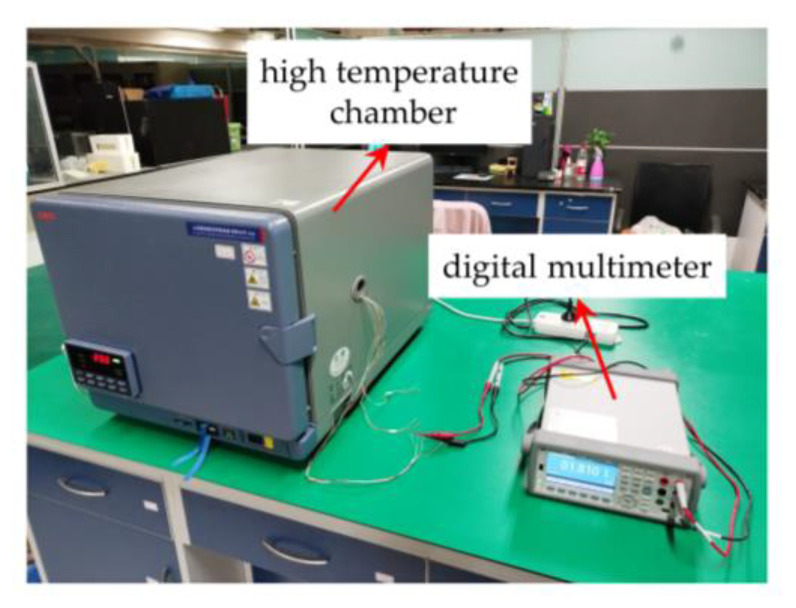
Experimental setup for testing resistances.

The reliability of the fabricated leads was verified by testing each resistance value of the Wheatstone bridge at 200 °C for a long time, as shown in Figure 8.

**Figure 9:**
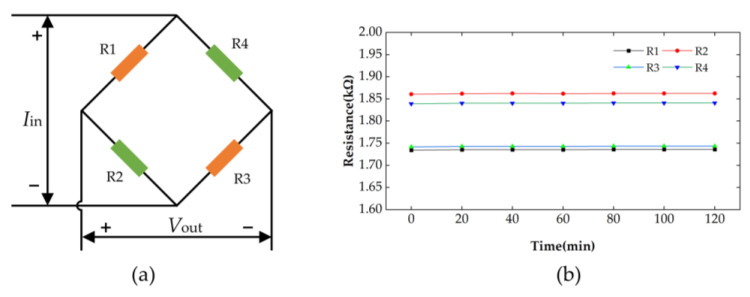
(**a**) The Wheatstone bridge. (**b**) Curve of the resistance variation within 2 h.

The sensor was placed in a high-temperature chamber and then heated up to 200 °C, followed by holding for 1 h. Finally, four resistance values of the Wheatstone bridge were recorded every 20 min for a total of 2 h, and the final test results are shown in Figure 9b. R1, R2, R3 and R4 in Figure 9a represent the four piezoresistors on the pressure sensor chip. It can be observed from Figure 9b that the resistance curves of R1, R2, R3 and R4 were basically stable. The standard deviations of R1, R2, R3 and R4 were 0.00059 kΩ, 0.00064 kΩ, 0.00058 kΩ and 0.00051 kΩ, respectively, which shows that the multilayer leads have high long-term reliability at high temperatures.

**Figure 10:**
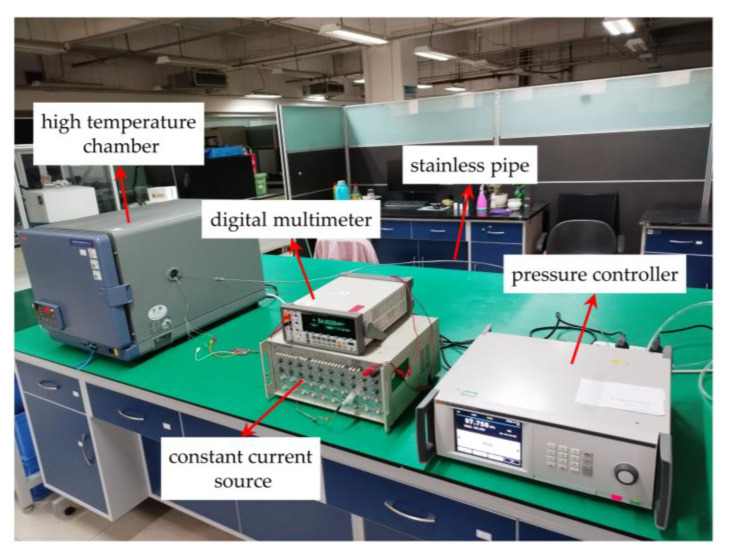
Experimental system for testing at different temperatures.

The static performance of the packaged pressure sensor was tested at room temperature and high temperatures, as shown in Figure 10.

The standard pressure load was provided by a pressure controller (6270A, Fluke, Everett, WA, USA) with a measurement range from 3.45 to 357 kPa. The lower limit of the standard pressure value that this pressure controller can easily provide is 4 kPa when the sensor is tested. Therefore, to facilitate the testing of the sensor, the minimum standard pressure value was taken to be 4 kPa. The multi-channel constant current source supplied the sensor with a constant current of 1 mA, and the voltage output signal of the sensor was read by a digital multimeter (8846A, Fluke, Everett, WA, USA). The high-temperature chamber (STH120, ESPEC, Osaka, Japan) provided a constant temperature environment. The pressure controller was connected to the sensor in the high-temperature chamber through a thin stainless pipe to test the performance of the sensor at different temperatures.

Room temperature, 50 °C, 100 °C, 150 °C and 200 °C were selected as the temperature points for testing. The range of the sensor designed in this paper was 0–250 kPa, so 4 kPa, 31 kPa, 62 kPa, 93 kPa, 124 kPa, 155 kPa, 186 kPa, 217 kPa and 250 kPa were selected as the pressure points for testing. The sensor was tested at 4 kPa; then, the pressure load was increased sequentially and tested at the remaining pressure points. This process is called forward travel. Conversely, it is called reverse travel. A cycle test consists of forward travel and reverse travel. In order to obtain more accurate test results, two cycles were carried out for the sensor at each temperature point. Finally, based on the test data at different temperatures, the performance indicators of the sensors were calculated.

**Figure 11:**
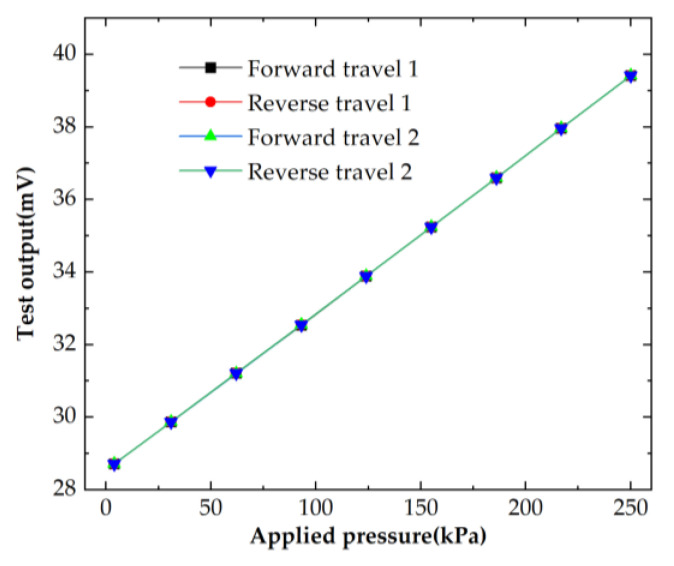
Output voltage of the sensor at room temperature.

The voltage output of the pressure sensor at room temperature (23 °C) is shown in Figure 11, where it can be observed that the output curves for all travels largely coincided. By calculation, the proposed sensor achieved a high accuracy of 0.277% FS (full scale), a nonlinearity of 0.265% FS, a low hysteresis of 0.047% FS, a high repeatability of 0.062% FS and a sensitivity of 0.043 mV/kPa/mA. Figure 11 shows that the sensor had a high zero output. This is mainly because the sensor chip used silicon leads, but the resistance of the silicon leads was not consistent, thus causing the Wheatstone bridge to be unbalanced. In the future, the shape of the silicon leads can be optimized to achieve a consistent resistance of the silicon leads, thus reducing the zero output.

The full-scale outputs of the proposed sensor and sensors in [13,14,27] were converted to the full-scale outputs under a constant voltage of 1 V. Then, the contrast in the full-scale outputs between them is displayed in Table 2. It can be observed that the full-scale output of the proposed sensor was only lower than the SiC sensor in [27]. However, the proposed SOI sensor has the advantage of mature processing technology and low cost. Therefore, despite the high response frequency of the proposed structure, the full-scale output of the sensor was not reduced much, which ensures the sensitivity.

**Figure 12:**
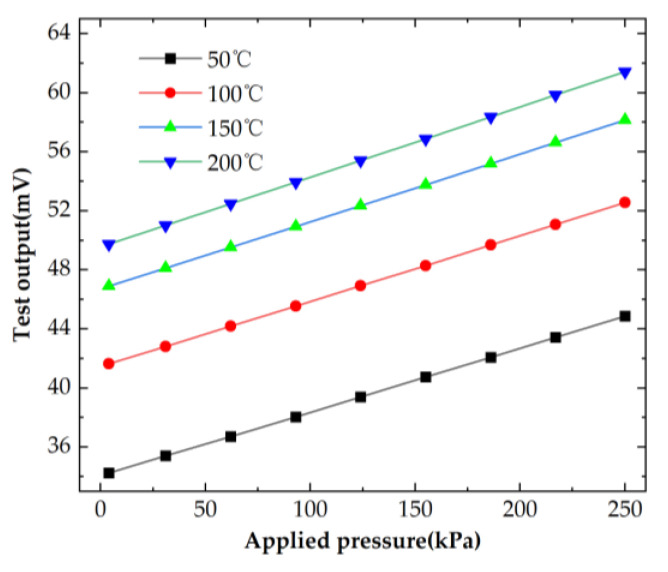
Output voltage of the sensor at different temperatures.

The outputs of the sensor at 50 °C, 100 °C, 150 °C and 200 °C are shown in Figure 12. It can be observed that there was a drift in the output of the sensor as the temperature increased. By calculation, the zero-point drift was 1.11% FS/°C, and the sensitivity drift was 0.05% FS/°C between room temperature and 200 °C. It can be observed from Table 2 that the sensitivity drift of the proposed sensor was smallest. The temperature coefficients of four piezoresistors were inconsistent, causing the zero drift to be a little large, but the zero drift was easily compensated. The piezoresistors were heavily doped so that the piezoresistive coefficient was less affected by the temperature, which caused the sensitivity drift to be very small. The sensor accuracy was 0.241% FS, the nonlinearity was 0.180% FS, the low hysteresis was 0.086% FS, the high repeatability was 0.137% FS and a sensitivity of 0.047 mV/kPa/mA was achieved at 200 °C. It can be seen from Table 2 that the accuracy of the proposed sensor was high. Therefore, the proposed sensor is still able to maintain high-performance indicators at high temperatures.

## 5. Conclusions

This paper designs a piezoresistive pressure sensor with the optimized crossbeam membrane, suitable for high temperatures. SOI was chosen as the sensor chip to solve the problem of leakage occurring at high temperatures in conventional silicon piezoresistive pressure sensors, while the sensor structure was designed to have the advantage of high-frequency response. A theoretical formulation of the mechanical performance with respect to the structural dimension was determined by a combination of curve fitting and the finite element. The optimal dimensions of the sensor structure were determined by solving the optimization model with MATLAB R2020a. The sensor chip was fabricated, packaged and tested, and the experimental results show that the sensor lead was very reliable and that the sensor had a good performance at 200 °C. For a pressure measurement range of 0–250 kPa, the accuracy was 0.241% FS, the nonlinearity was 0.180% FS, the low hysteresis was 0.086% FS and the high repeatability was 0.137% FS. Therefore, for pressure measurements at high temperatures, the pressure sensor designed in this paper has broad application prospects in aerospace.

## Figures and Tables

**Table 1:** Final dimensional variable values.

Parameters	*L*	*H*	*b*_1_	*b*_2_	*a*	*h*
Dimension (μm)	1100	25	280	700	40	4

**Table 2:** Comparison with other sensors.

Sensor	Full Range Pressure (kPa)	Full Scale Output (mV/V)	Sensitivity Drift (% FS/°C)	Accuracy (% FS)
Proposed sensor	0–250	7.75	0.05	0.241
Sensor in [13]	0–180	6.48	0.45	>0.476
Sensor in [14]	0–100	4.5	−0.19	0.172
Sensor in [27]	10 MPa	15.6	−0.134	0.29

## Data Availability

Data sharing is not applicable to this article.
